# Host-Based Th2 Cell Therapy for Prolongation of Cardiac Allograft Viability

**DOI:** 10.1371/journal.pone.0018885

**Published:** 2011-04-29

**Authors:** Shoba Amarnath, Hao Chen, Jason E. Foley, Carliann M. Costanzo, Joel D. Sennesh, Michael A. Solomon, Daniel H. Fowler

**Affiliations:** 1 Experimental Transplantation and Immunology Branch, National Cancer Institute, National Institutes of Health, Bethesda, Maryland, United States of America; 2 Critical Care Medicine, Clinical Center, National Institutes of Health, Bethesda, Maryland, United States of America; 3 Department of Cardiac Surgery, Huadong Hospital, Fudan University, Shanghai, China; 4 Department of Pathology, Inova Fairfax Hospital, Fairfax, Virginia, United States of America; 5 Cardiovascular and Pulmonary Branch, National Heart, Lung and Blood Institute, National Institutes of Health, Bethesda, Maryland, United States of America; University of California Los Angeles, United States of America

## Abstract

Donor T cell transfusion, which is a long-standing approach to prevent allograft rejection, operates indirectly by alteration of host T cell immunity. We therefore hypothesized that adoptive transfer of immune regulatory host Th2 cells would represent a novel intervention to enhance cardiac allograft survival. Using a well-described rat cardiac transplant model, we first developed a method for ex vivo manufacture of rat host-type Th2 cells in rapamycin, with subsequent injection of such Th2.R cells prior to class I and class II disparate cardiac allografting. Second, we determined whether Th2.R cell transfer polarized host immunity towards a Th2 phenotype. And third, we evaluated whether Th2.R cell therapy prolonged allograft viability when used alone or in combination with a short-course of cyclosporine (CSA) therapy. We found that host-type Th2.R cell therapy prior to cardiac allografting: (1) reduced the frequency of activated T cells in secondary lymphoid organs; (2) shifted post-transplant cytokines towards a Th2 phenotype; and (3) prolonged allograft viability when used in combination with short-course CSA therapy. These results provide further support for the rationale to use “direct” host T cell therapy for prolongation of allograft viability as an alternative to “indirect” therapy mediated by donor T cell infusion.

## Introduction

Clinical interventions to prolong cardiac allograft survival have relied primarily on long-term post-transplant administration of calcineurin inhibitors such as cyclosporine A (CSA) for suppression of host T cells that mediate rejection [1] [reviewed in [2]]. However, long-term calcineurin inhibitor therapy is typically only partially effective and the T cell immune deficiency predisposes to life threatening infection and malignancy [reviewed in [3]]. As such, new approaches in transplantation seek to limit patient exposure to calcineurin inhibitors and to promote immune tolerance through either pharmacologic or cellular interventions [reviewed in [4], [5]].

“Donor specific tolerance” was observed when recipients of T cell-containing, third-party blood transfusion prior to clinical organ transplantation were found to have a reduced incidence of graft rejection [6], [7], [8]. Recent animal model experiments have demonstrated that the reduction in graft rejection through donor T cell infusion occurs “indirectly” through modulation of host T cells [9], [10]. Most recently, in a murine model of transplantation tolerance, donor regulatory T (Treg) cells contained within transferred blood products were found to induce naive host T cells to adopt a Treg phenotype [11].

As such, various T cell transfer methods that result in the modulation of host T cell populations represent a general approach to prolong allograft survival. Recently, we have shown that donor T cells polarized into a Th2 phenotype modulate host T cells towards a Th2 phenotype, thereby preventing graft rejection in a murine model of hematopoietic stem cell transplantation [12], [13]. Based on this background, we now project that host Th2 cell adoptive transfer may represent a “direct” pathway to prolong solid organ allograft viability. Host T cell therapy would be particularly useful for cardiac allograft recipients due to the lack of cadaveric donor T cells. In addition, rat cardiac allograft rejection has been characterized as a Th1-type process [14], and therefore predictably amenable to Th2 cell therapy, which we have shown to be capable of modulating Th1-type transplantation responses [12]. Towards this aim, we tested our hypothesis in a well-characterized rat cardiac allograft transplantation model.

In murine models of graft rejection [12] and graft-versus-host disease [15], we found that adoptive transfer of Th2 cells that were manufactured ex vivo in rapamycin (“Th2.R cells”) were more effective than control Th2 cells; the increased in vivo efficacy of Th2.R cells is likely due to their rapamycin-induced anti-apoptotic phenotype, which permits prolonged in vivo T cell persistence [13]. In light of these data, we hypothesized that adoptive cell therapy using host-type Th2.R cells may represent a novel approach to modulate host immunity towards a Th2 phenotype for prolongation of solid organ transplant survival.

## Results

### Ex vivo manufacture of rat CD4^+^ Th2 cells with or without rapamycin

**Figure 1:**
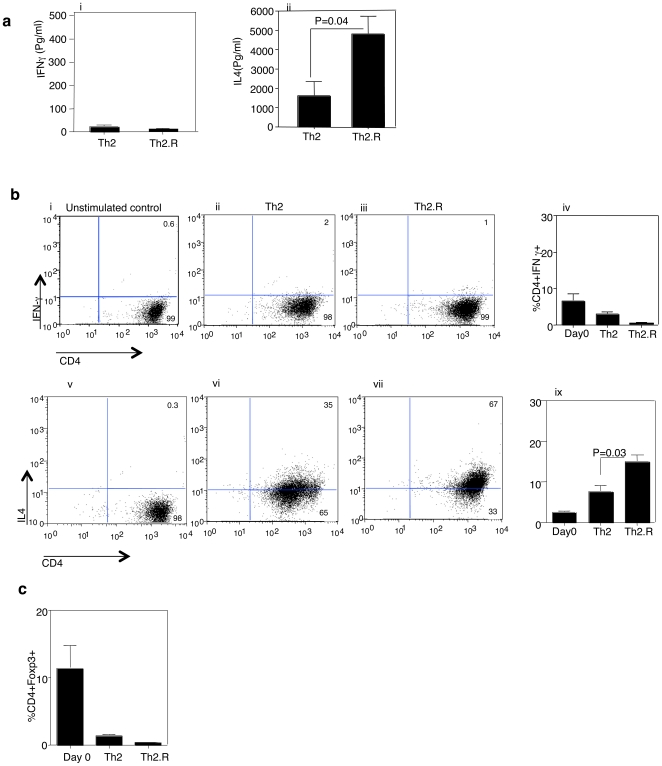
Characterization of rat Th2 cells. CD4^+^ T cells were isolated and expanded using anti-CD3 and anti-CD28 co-stimulation in the presence of rhIL-2, rrIL-4, and rhIL-7 either with rapamycin (“Th2.R”) or without rapamycin (“Th2”) for 3 days. (a) Expanded Th2 and Th2.R cells were subjected to repeat co-stimulation, and the 24 h supernatant was tested for cytokine content using multiplex bead array. (b) After repeat co-stimulation, Th2 and Th2.R cells were evaluated for intra-cellular expression of IFN-γ and IL-4 by flow cytometry. (c) On day 3, cells were harvested and intra-cellular flow cytometry was performed to evaluate Foxp3 transcription factor expression. Results are a summary of n = 10 cultures.

There are no reports in the literature pertaining to the ex vivo manufacture of rat Th2 cells in the presence of rapamycin; as such, we first evaluated if rat CD4^+^ T cells could be polarized to a Th2 phenotype during rapamycin exposure. In previous experiments evaluating Th2 cell therapy in the context of murine allogeneic bone marrow transplantation, we identified an effective strategy whereby cytokine polarization occurred ex vivo in a polyclonal manner, with subsequent acquisition of allosensitization in vivo [16]; as such, for these studies, we performed cytokine polarization in the context of polyclonal co-stimulation. Co-stimulation and IL-4 priming in the presence or absence of rapamycin resulted in T cells expressing a Th2 phenotype, as defined by minimal IFN-γ secretion (Fig. 1a, panel i; Fig. 1b, panels i, ii, iii) and high levels of IL-4 secretion (Fig. 1a, panel ii; Fig. 1b, panel iv, v, vi). Relative to control Th2 cells (“Th2”), rapamycin treated Th2 cells (“Th2.R”) secreted higher amounts of IL-4 (P<0.05 for supernatant assay in Fig. 1a; p = 0.03 for i.c. flow cytometry assay in Fig. 1b). Control Th2 cells and Th2.R cells each had minimal expression of the Treg transcription factor, Foxp3 (Fig. 1c; percent CD4^+^Foxp3^+^ expression, mean ± SEM, Th2 vs. Th2.R: 2.0±0.5 vs. 0.5±0.6). As such, ex vivo rapamycin enhanced host T cell Th2 polarity without promoting a Treg phenotype.

### Th2.R cell therapy combined with CSA therapy reduces T cell activation during allograft challenge and augments post-transplant Th2 polarity

**Figure 2:**
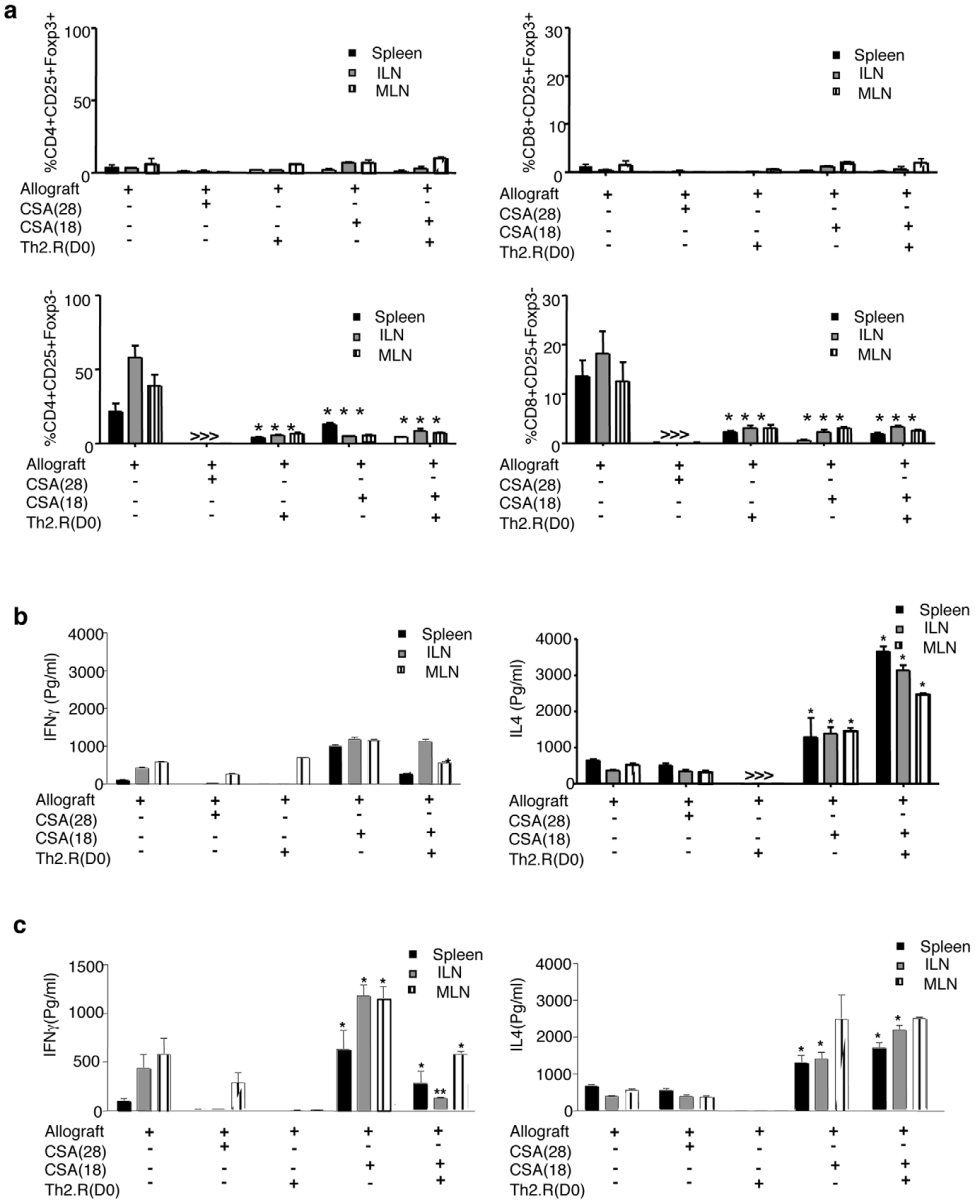
Host Th2.R cell infusion prolongs cardiac allograft survival. Rat allogeneic cardiac transplants was performed and assigned to one of five treatment cohorts, including: no drug and no cell therapy (“rejection control”); 28-day daily cyclosporine A therapy [“CSA(28);” engraftment control]; Th2.R adoptive cell therapy alone on the day of transplant [“Th2.R(d0)”]; 18-day, short-course CSA therapy alone [“CSA(18)”; experimental control]; or a combination of short-course CSA plus Th2.R cell therapy [“CSA(18)+Th2.R(d0)”]. (a) At day 28 post-transplant, recipients were euthanized and spleen, inguinal lymph nodes, and mesenteric lymph nodes were harvested. The frequency of activated CD4^+^ and CD8^+^ T cells in each cohort at each organ site was then determined by flow cytometry (percent of CD4^+^ or CD8^+^ T cells that co-expressed CD25 in the absence of Foxp3 expression; results are mean ± SEM of 3 evaluated per cohort; *, indicates p≤0.05; **, indicates p≤0.005). Cells harvested at day 28 post-transplant were co-stimulated (b) or allo-stimulated (c & d), and the 24 h supernatants were tested for cytokine content by multiplex assay.

Expanded host-type Th2.R cells were infused intravenously into recipient (host-type) rats following cardiac transplantation surgery at day 0. Cohorts received the allograft either alone (“allograft”) or in combination with: CSA for 28 days (CSA 28); CSA for 18 days (CSA 18); Th2.R cells; or Th2.R cells plus (CSA 18). At the final day 28 post-transplant endpoint, recipients in the rejection control cohort that did not receive CSA or Th2.R cells had low frequencies of CD4^+^CD25^+^Foxp3^+^ T cells (Fig. 2a top panels) and had high frequencies of CD4^+^CD25^+^Foxp3^−^ activated effector T cells (Fig. 2a, bottom left panel) and CD8^+^CD25^+^Foxp3^−^ activated effector T cells (Fig. 2a, bottom right panel) in the spleen, inguinal lymph nodes, and mesenteric lymph nodes. By comparison, recipients of daily subcutaneous CSA therapy had undetectable levels of activated effector CD4^+^ and CD8^+^ T cells. Statistical analyses were performed, with comparison of each experimental cohort to the rejection control cohort. Relative to rejection controls, recipients of a short-course of CSA alone, Th2.R cells alone, or the combination of short-course CSA plus Th2.R cells had decreased frequencies of effector CD4^+^ and CD8^+^ T cells; as such, both CSA and Th2.R cell infusion reduced in vivo host T cell activation during allograft challenge.

Next, we evaluated the cytokine profile of the various cohorts at day 28 post-transplant; post-transplant T cell cytokine production was induced after polyclonal stimulation using co-stimulation (Fig. 2b) and by syngeneic and allogeneic antigen-presenting-cell (APC) stimulation to determine allospecific cytokine secretion (Fig. 2c). Upon co-stimulation and allogeneic APC activation, spleen and lymphoid cells isolated from recipients of short-course CSA or the combination of short-course CSA plus Th2.R cells had increased secretion of IL-4 relative to spleen and lymphoid cells isolated from recipients of either continuous daily CSA therapy or Th2.R cell therapy alone (Fig. 2b and 2c, respectively); the mechanism(s) whereby short-course CSA consistently increased post-transplant IL-4 secretion capacity is not clear and was not addressed in our experiments. The rejection control cohort secreted low levels of IL-4. As such, whereas daily CSA therapy suppressed the host Th2 response, short-course CSA and Th2 cell transfer promoted Th2 immunity. Furthermore, infusion of Th2.R cells in the context of short-course CSA therapy reduced post-transplant T cell capacity to secrete IFN-γ in both a polyclonal and an allospecific manner (Fig. 2b and 2c, respectively).

### Evaluation of Th2.R cell therapy timing and rapamycin co-administration

**Figure 3:**
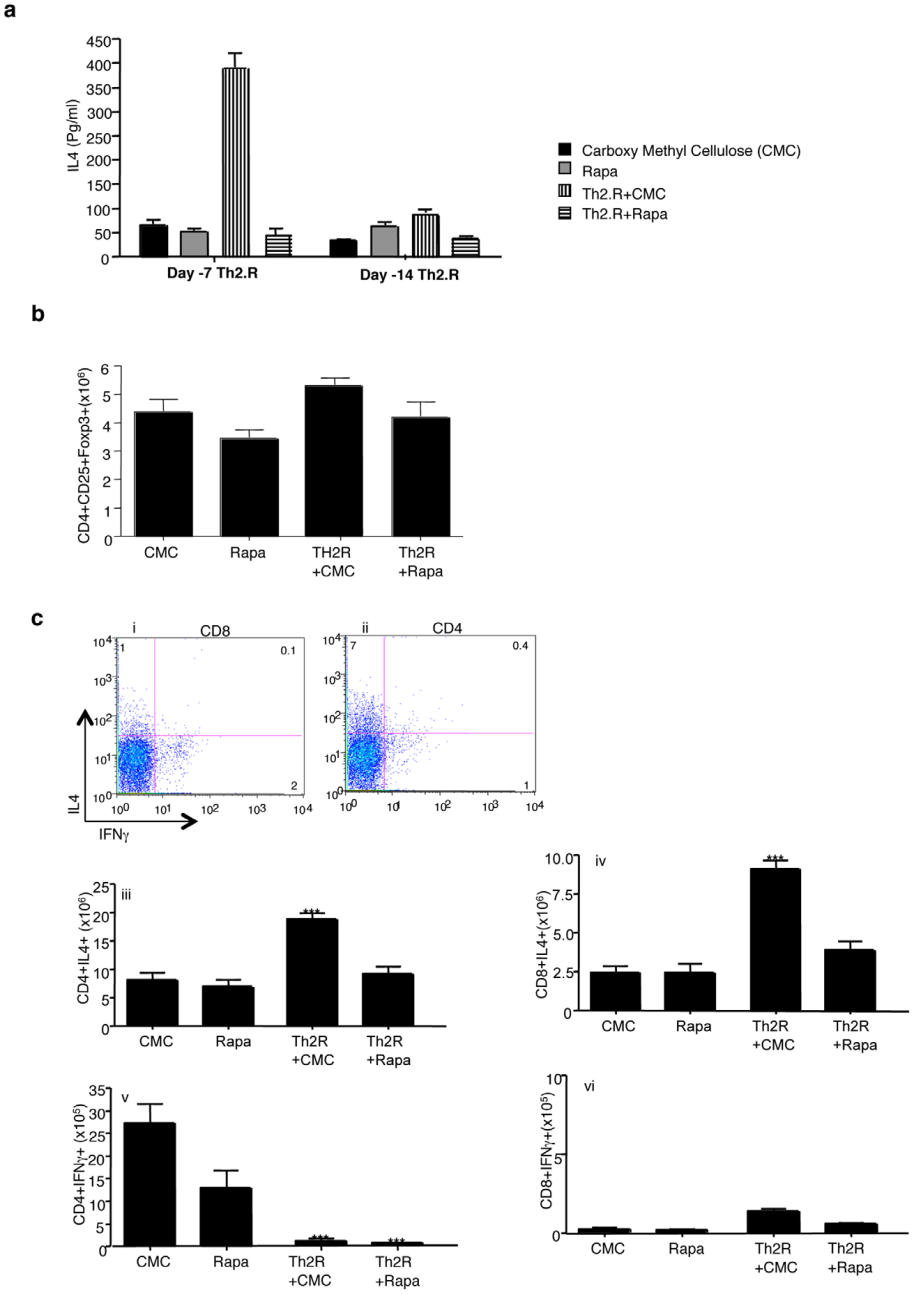
Evaluation of Th2.R cell timing and rapamycin co-administration. Host-type BN rats received an intravenous infusion of isogeneic Th2.R cells either at day −7 or day −14 prior to host euthanasia at “day 0”, with subsequent immune evaluation; Th2 cell therapy was administered either alone or in combination with in vivo rapamycin therapy. (a) Harvested spleen, inguinal lymph node, and mesenteric lymph node cells were harvested and subjected to co-stimulation; resultant 24 h supernatants were then tested for cytokine content by Multiplex assay. (b) % Foxp3 expression was measured using IC flow on splenocytes at day7 (c) After co-stimulation, CD4^+^ and CD8^+^ T cells from recipients in each cohort were evaluated by intra-cellular flow cytometry for IFN-γ and IL-4 production. * indicates P<0.05; n = 5 per cohort.

Further experiments were performed in host-type BN rats to optimize Th2.R cell therapy. We hypothesized that infusion of host Th2.R cells one or two weeks prior to the allogeneic transplant might induce more marked host skewing towards a Th2 state, thereby enhancing a Th2.R cell protection against graft rejection. In lieu with our prior finding that murine Th2.R cells were relatively resistant to in vivo rapamycin drug therapy [17], we also hypothesized that pre-transplant co-administration of rapamycin might further augment the host Th2 state. Indeed, at day 7 after host Th2.R cell infusion, there was a dramatic increase in host capacity to secrete IL-4 (Fig. 3a); however, co-administration of rapamycin actually abrogated this host Th2 shift. Of note, host immunity was not significantly polarized at day 14 after Th2.R cell infusion; as such, further experiments focused on an anti-rejection strategy incorporating Th2.R cell infusion at day −7 pre-transplant. Seven days after treatment with CD4^+^ Th2.R cells, no significant increase in Foxp3^+^ T cells was observed (Fig. 3b). Both host CD4^+^ and CD8^+^ T cells were enriched for IL-4 secretion capacity (Fig. 3c; Representative flow plot panel i gated on CD8^+^ T cells and ii gated on CD4^+^ T cells; summary iii and iv); as such, similar to our previous work in murine models, the adoptive transfer of highly purified rat CD4^+^ Th2.R cells resulted in the transfer of Th2 immunity to other host T cell populations. Of note, Th2.R cell induction of host CD4^+^ and CD8^+^ T cell IL-4 secretion was inhibited by rapamycin co-administration. Th2 skewing was confirmed by the ability of the host CD8+ T cells to secrete IL4. Furthermore, seven days after host Th2.R cell infusion, we observed a marked decrease in host CD4^+^ T cell capacity for IFN-γ secretion (Fig. 3c; v). Interestingly, rapamycin drug therapy did not reduce CD4^+^ T cell IFN-γ secretion in a statistically significant manner. In addition, Th2.R cells or rapamycin did not significantly alter IFN-γ secretion capacity within host CD8^+^ T cells (Fig. 3c; vi). Since rapamycin was not permissive for Th2 polarization in our system, further experiments tested Th2.R cell therapy in the setting of CSA rather than rapamycin drug therapy.

### Th2.R cell therapy combined with short-course CSA therapy prolongs cardiac allograft viability

**Figure 4:**
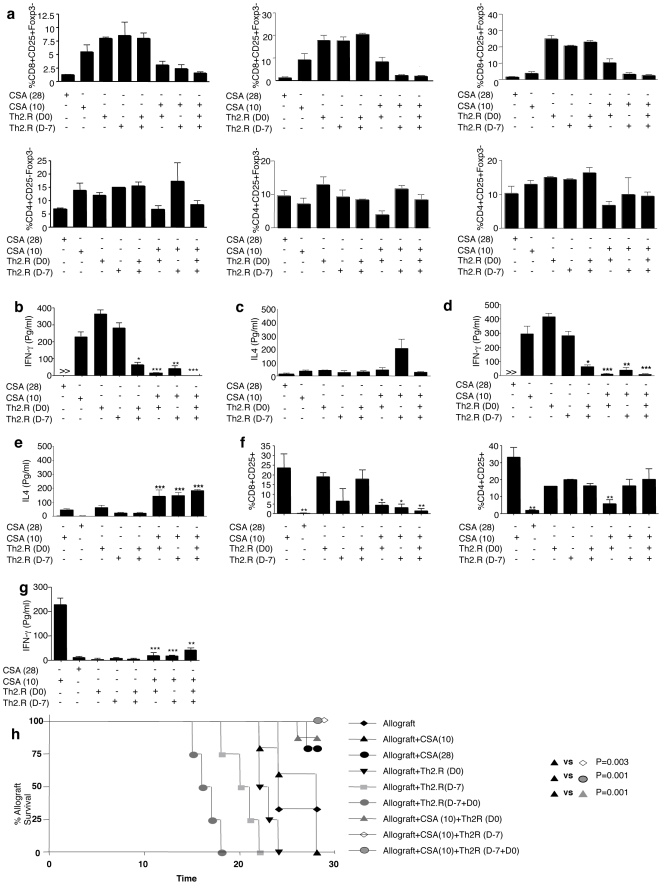
Th2.R cell therapy plus cyclosporine reduces host T cell activation, induces a host Th2 phenotype, and reduces intra-cardiac allospecific T cells. Rat allogeneic cardiac transplants were performed and assigned to one of eight treatment cohorts, including; 28-day daily cyclosporine A therapy [“CSA(28)”; engraftment control]; Th2.R adoptive cell therapy alone (“Th2.R”) at day 0 (D0), day-7 (D-7) or both (D-7+D0); 10-day, short-course CSA therapy alone [“CSA(10);” experimental control]; or a combination of short-course CSA plus Th2.R cell therapy [“CSA(10)+Th2.R”] at day 0 (D0), day-7 (D-7) or both (D-7+D0). (a) At day 28 post-transplant, transplant recipients were euthanized and splenocytes were harvested. The frequency of activated CD4^+^ and CD8^+^ T cells in each cohort at each organ site was then determined by flow cytometry (percent of CD4^+^ or CD8^+^ T cells that co-expressed CD25 in the absence of Foxp3 expression; results are mean ± SEM of 5 or 7 evaluated per cohort; *, indicates p<0.05). Splenocytes were harvested and subjected to either co-stimulation (b & c) or syngeneic and allogeneic APC stimulation (d & e; allospecific cytokine secretion is shown); resultant 24 h supernatants were then tested for cytokine content by Multiplex assay. (f) The frequency of activated CD4^+^ and CD8^+^ T cells in harvested cardiac tissue was determined by flow cytometry (percent of CD4^+^ or CD8^+^ T cells that co-expressed CD25 in the absence of Foxp3 expression; results are mean ± SEM of 7 evaluated per cohort; *, indicates p<0.05). (g) Intracardiac T cells were subjected to stimulation with allogeneic dendritic cells; resultant 24 h supernatants were then tested for cytokine content by Multiplex assay. (h) Survival of cardiac allografts between various cohorts is shown.

We next evaluated host-based Th2.R cell therapy in a stringent, 28-day model of allograft rejection that utilized CSA for only the initial 10 days post-transplant. At day 28 post-transplant, as anticipated, recipients of daily CSA (through day 28 post-transplant) had reduced CD8^+^ T cell activation in the spleen, inguinal lymph nodes, and mesenteric lymph nodes relative to recipients of short-course CSA (Fig. 4a; top panels). Remarkably, host Th2.R cell infusion plus short-course CSA also reduced CD8^+^ T cell activation at these tissue sites to levels comparable with daily CSA therapy; in general, this CD8^+^ T cell inhibition occurred whether Th2.R cells were administered at day −7, day 0, or day −7 plus day 0. However, Th2.R cell infusion without short-course CSA did not inhibit CD8^+^ T cell activation. In marked contrast to these CD8^+^ T cell results, CSA therapy and/or Th2.R cell therapy did not significantly alter CD4^+^ T cell activation (Fig. 4a; bottom panels); it is possible that the substantial CD4^+^ T cell activation in Th2.R cell recipients reflects activation of the adoptively transferred Th2.R cells.

Next, we evaluated the in vivo cytokine profile at day 28 post-transplant. Spleen cells from recipients of daily CSA therapy secreted very low levels of IFN-γ in a polyclonal manner (Fig. 4b) or in an allospecific manner (Fig. 4d). In contrast, spleen cells from recipients of either short-course CSA alone or Th2.R cells alone had increased polyclonal and allospecific secretion of IFN-γ. Of note, multiple infusions of Th2.R cells (both day −7 and day 0) prior to transplantation resulted in a decrease in IFN-γ secretion relative to recipients of a single dose Th2.R cells. Recipients of short-course CSA plus Th2.R cells (independent to Th2.R cell timing) had decreased polyclonal and allospecific IFN-γ secretion relative to recipients of short-course CSA alone. Relative to recipient of short-course CSA alone, recipients of short-course CSA plus Th2.R cell infusion had an increase in allospecific IL-4 secretion (Fig. 4e); this increase in allospecific IL-4 secretion was observed in each of the three cohorts to receive Th2.R cells. Interestingly, limited differences was noted in IL-4 secretion between cohorts on polyclonal stimulation (Fig. 4c).

Furthermore, at day 28 post-transplant, we tested for the presence of activated T cells within the cardiac allografts. As anticipated, recipients of short-course CSA alone had a high frequency of activated CD4^+^ and CD8^+^ T cells infiltrating the graft (Fig. 4f); by comparison, recipients of daily CSA had minimal evidence of intra-cardiac activated T cells. Relative to recipients of short-course CSA alone, recipients of Th2.R cell therapy in combination with short-course CSA therapy had reduced frequencies of intra-cardiac activated CD8^+^ T cells. Interestingly, Th2.R cell therapy reduction in the frequency of intra-graft activated CD4^+^ T cells was modest relative to CD8^+^ T cells; however, marked reductions of intra-graft activated CD4^+^ T cells were observed in recipients of Th2.R cells on day 0 of transplant. Most importantly, intra-cardiac T cells from Th2.R cell recipients had greatly reduced capacity to secrete IFN-γ in an allospecific manner relative to recipients of short-course CSA (Fig. 4g).

**Figure 5:**
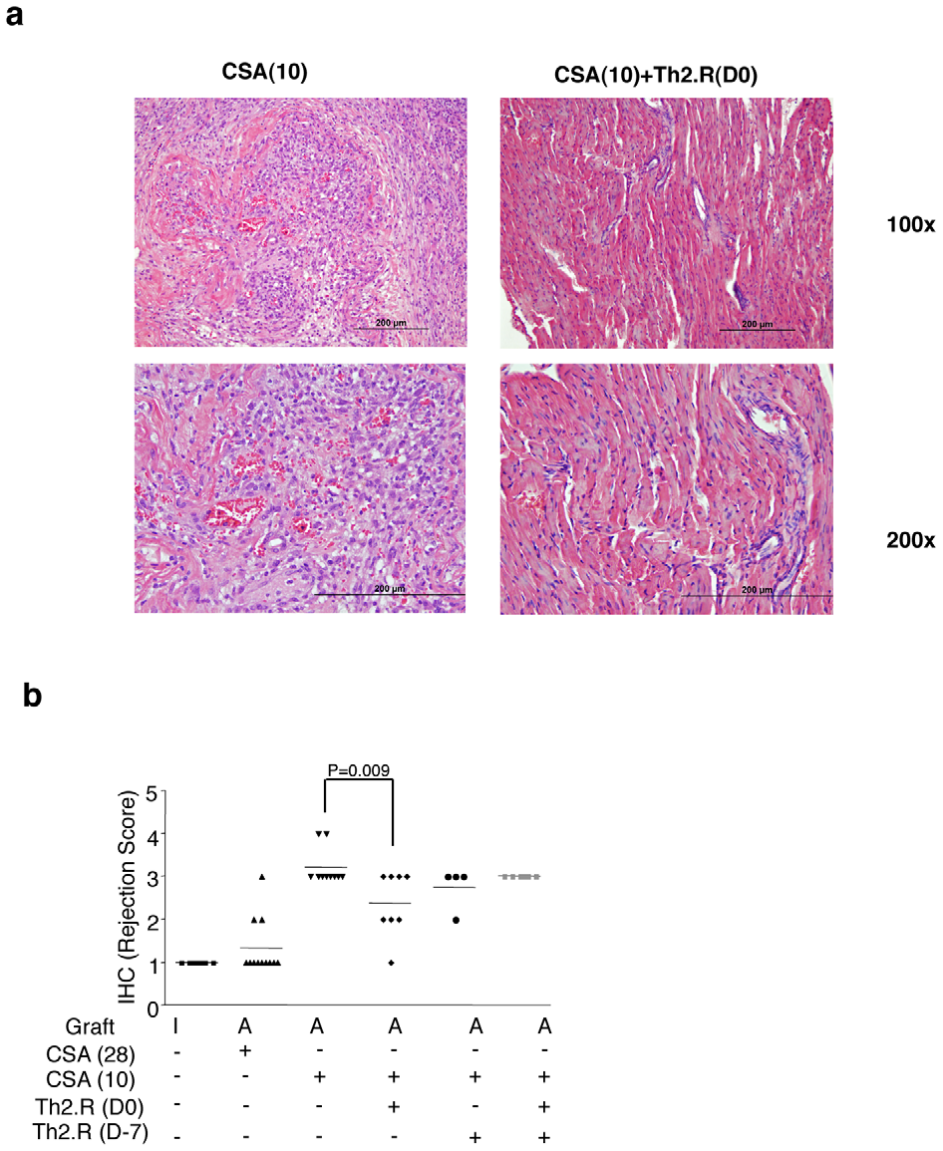
Th2.R cell therapy plus cyclosporine prolongs cardiac allograft survival. Allograft recipients were monitored for cardiac viability through the 28-day post-transplant period of observation. Rejection and engraftment control cohorts received a 10-day short-course of cyclosporine A [“CSA(10]” or a daily 28-day course of “CSA [CSA(28)”]. Other cohorts received either no cyclosporine A or short-course CSA(10) in combination with Th2.R cell therapy at day −7 [“Th2.R(D-7)” or “CSA(10)+Th2.R(D-7)”], day 0 [“Th2.R(D0)” or “CSA(10)+Th2.R(D0)”], or day −7 plus day 0 [“Th2.R(D-7+D0)” or “CSA(10)+Th2.R(D-7+DO)”]. (a) Hematoxylin and eosin staining was performed at day 28 post-transplant. Left panels show a representative example of severe allograft rejection in recipients of short-course CSA(10) alone (characterized by diffuse inflammation and necrosis); right panels show a representative example of relatively preserved myocardial cell structure and reduced mononuclear cell infiltration in recipients of short-course CSA(10) therapy plus Th2.R cell therapy. (b) Cumulative histology score between various cohorts is shown. I = isograft, A = Allograft; IHC score = Immunohistochemistry score.

As anticipated, the control group that received short-course CSA universally had non-viable cardiac allografts by day 28 post-transplant (Fig. 4h); recipients of Th2.R cells alone also universally had nonviable allografts. Administration of daily CSA predictably increased the incidence of clinical graft viability (graft viability frequency, 85%). Remarkably, recipients of short-course CSA combined with Th2.R cells at day 0, day −7, or day −7 plus day 0 were also generally protected from graft failure (graft viability frequencies: 86%, 100%, 100%, respectively; Table 1). Haematoxylin and eosin staining obtained at day 28 post-transplant demonstrated that recipients of short-course CSA, who had poor allograft viability, had severe myocardial necrosis and a dense mononuclear cell infiltration (Fig. 5a). In contrast, recipients of short-course CSA combined with host Th2.R cell therapy had minimal myocardial necrosis in the setting of substantial mononuclear cell infiltration; importantly, recipients of short-course CSA plus Th2 cells on day 0 of transplant had a statistically significant reduction in rejection by standard histology criteria (Fig. 5b). Of note, recipients of Th2 cells at day −7 plus day 0 of transplant or day −7 of transplant, who had 100% graft viability, did not have reduced histology scores.

**Table 1:** Summary of experimental design.

Experimental Design for data in Figure #2			
Treatment Cohorts (n)	Cardiac Allograft^1^	CSA Drug Therapy (Total Days)^2^	ImmunotherapyTh2.R (Day administered)^3^
1. n = 3 (Rejection Control)	Y	N	N
2. n = 3 (Treatment Control)	Y	Y (28)	N
3. n = 3 (Sub-optimal Drug Therapy)	Y	Y (18)	N
4. n = 3 (Immunotherapy)	Y	N	Y (0)
5. n = 3 (Immunotherapy)	Y	Y (18)	Y (0)

Experimental Design for data in Figure #3			
Treatment Cohorts (n)	Cardiac Allograft	Rapamycin Drug Therapy (Total Days prior to transplant)^4^	ImmunotherapyTh2.R (Day administered)
1(Experimental Control), n = 5	N	CMC (7)	N
2(Experimental Control), n = 5	N	RAPA (7)	N
3(Immunotherapy), n = 5	N	CMC (7)	Y (−7)
4(Immunotherapy), n = 5	N	RAPA (7)	Y (−7)
5(Experimental Control), n = 5	N	CMC (14)	N
6(Experimental Control), n = 5	N	RAPA (14)	N
7(Immunotherapy), n = 5	N	CMC (14)	Y (−14)
8(Immunotherapy), n = 5	N	RAPA (14)	Y (−14)

Experimental Design for data in Figure #4&5			
Treatment Cohorts	Cardiac Allograft	CSA Drug Therapy (Total Days)	ImmunotherapyTh2.R (Day administered)
1(Rejection Control), n = 7	Y	Y (10)	N
2(Treatment Control), n = 7	Y	Y (28)	N
3(Immunotherapy), n = 7	Y	N	Y (0)
4(Immunotherapy), n = 5	Y	N	Y (−7)
5(Immunotherapy), n = 4	Y	N	Y (0 & −7)
6(Immunotherapy), n = 5	Y	Y (10)	Y (0)
7(Immunotherapy), n = 7	Y	Y (10)	Y (−7)
8(Immunotherapy), n = 7	Y	Y (10)	Y (0 & −7)

1 Y = yes, N = no; DA donor hearts transplanted into BN recipient rats.

2 Cyclosporine (CSA) administered subcutaneously (10 mg/kg/day).

3 Th2.R = Th2 cells were generated from host BN CD4+ T cells.

4 Rapamycin (Rapa) administered i.p (1.5 mg/kg/day); CMC = carboxymethylcellulose.

## Discussion

Utilizing a well-characterized experimental rat cardiac transplantation model, we have shown that allograft viability can be prolonged by direct modulation of host immunity via adoptive T cell therapy using rapamycin-generated Th2-type cells. These data add to the growing body of information relating to cellular approaches to rejection prevention, and squarely places an emphasis upon use of host T cell populations as an alternative to indirect modulation of host immunity via third-party donor cell infusions. Importantly, as a step towards potential clinical translation, we have determined that host Th2-type cell adoptive transfer is efficacious when used in combination with calcineurin inhibitor therapy, which remains the standard of care therapy.

Reports more than 30 years ago determined that donor-specific [18] or third-party [19] transfusions reduced solid organ transplantation rejection through a phenomenon termed donor-specific-tolerance. However, further clinical advances with respect to use of adoptive cell therapy for rejection abrogation did not ensue, in part because the mechanism(s) underlying transfusion-induced tolerance have not been fully characterized and in part because adoptive cell therapy is only now becoming a burgeoning translational medical approach [20]. It has long been proposed that modulation of host T cell immunity might be harnessed to promote graft tolerance, including an early study of “host T cell vaccination” prior to experimental transplantation [21]; however, further investigations into host derived T cell based engraftment strategies have been limited. Extensive mechanistic data exists on donor specific tolerance [11], [14] where adoptive transfer of ex vivo generated host Treg cells prevents allograft rejection [22], [23]. Although focus has been placed upon host Tregs for cellular induction of transplantation tolerance [reviewed in [24], [25]], other host T cell populations such as CD4^−^CD8^−^ “double-negative” T cells have been shown to facilitate engraftment [26]. In this context, our current results using host-type, rapamycin-generated Th2 cells indicate that a diversity of functionally-defined host T cell subsets represent candidate populations to prevent solid organ graft rejection.

The current study is the first to demonstrate that the adoptive transfer of Th2 polarized host T cells represents a candidate approach to prolong cardiac allograft viability. Although enthusiasm for the potential role of Th2 responses for the regulation of Th1-mediated solid organ rejection has waned with the resurgence of Treg cell research [reviewed in [27]], several previous studies have supported a beneficial role of Th2 cells in cardiac transplantation models, including: evidence that type II cytokines help promote ‘infectious tolerance’ [28]; modulation of the Th1/Th2 balance via ICAM/LFA blockade [14]; and Treg cell up-regulation of IL-4 and Th2 responses during infectious tolerance [29]. In our studies, the phenotype of the ex vivo, rapamycin-generated host T cell population was consistent with an effector Th2 phenotype, as indicated by prolific secretion of IL-4, a high precursor frequency of cells capable of IL-4 secretion, and minimal expression of the Treg cell transcription factor, FoxP3. These results stand somewhat in contrast to other ex vivo results that described rapamycin to promote Treg cell differentiation [30], but are consistent with our murine results that have identified a capacity to generate either effector Th1 and Th2 populations in rapamycin depending upon polarizing cytokine exposure [17], [31].

Most importantly, the adoptively transferred T cells mediated a Th2-type response in vivo, as evidenced by: increased post-transfer IL-4 secretion; reduced post-transfer IFN-γ secretion; and minimal induction of Foxp3-expressing T cells. We also observed that recipients of the CD4^+^ Th2.R cells were enriched for post-transplant CD8^+^ T cell capacity for IL-4 secretion capacity; this result indicates that the post-transplant Th2 polarization induced through Th2.R cell infusion could be attributable not only to the infused Th2 cell product but also to the transfer of Th2 polarity to endogenous T cells. Similar to our previous observations in murine bone marrow transplant models that utilizing donor-derived Th2.R cells [15], [32], we also observed that host adoptive Th2 cell therapy significantly reduced the number of post-transplant CD8^+^ T cells in secondary lymphoid organs; given the major role for host CD8^+^ T cells in mediating cardiac allograft rejection [33], [34], we speculate that Th2-mediated control of CD8^+^ T cell immunity may account in part for allograft survival effect we observed in this model. It is interesting to note that Th2.R cell infusion alone did not achieve post-transplant skewing towards a Th2 phenotype, whereas Th2.R cell infusion plus short-course CSA consistently yielded increased post-transplant IL-4 and reduced post-transplant IFN-γ. We speculate that CSA therapy is limiting the expansion of the host Th1-type rejection response, thereby allowing expansion of the infused Th2 cells; in the absence of CSA, the Th2 cells may be inhibited by a predominant Th1 response.

Our results stand somewhat in contrast to previous studies that found Tc2 cells [35] or IL-4 [36] to be detrimental to graft survival. We speculate that our use of purified CD4^+^ T cells that secreted high levels of IL-4 may help account for the positive results we achieved with a type II cytokine strategy; further experiments will be required to clarify specific molecular mechanisms that account for the Th2.R cell therapeutic effect. It should be mentioned that our model utilizes a relatively short recipient follow-up for assessment of allograft viability, and as such, the effect of the Th2 cell strategy on long term allograft viability would require further study [37]. We observed that both the long-term immune suppression cohort and the short-course suppression plus Th2.R cohort each had prolonged allograft viability; therefore, from these experiments, we can not conclude that one strategy is advantageous relative to the other. Rather, we conclude that short-course suppression plus infusion of a T cell modulation population such as the host Th2.R cells may represent an alternative to long-term immune suppression. Determination of the relative risks and benefits of each approach would require further comparative studies in additional models.

It is important to point out that, in spite of the increase in cardiac viability in Th2.R cell recipients, such recipients did not have a reduction in the rejection score by standard histology criteria. It should be noted that the rejection score is based heavily on mononuclear cell infiltration; because we found that Th2.R cell recipients had relatively dense intra-cardiac T cell infiltration by cells that did not secrete allospecific IFN-γ, it is possible that in the context of interventions such as host-type Th2.R cell transfer, cardiac infiltration by mononuclear cells alone may not accurately predict rejection. Finally, it should also be noted that we did not assess the impact of Th2.R cell infusion on alloantibody formation, which can play an important role as a mediator of kidney allograft rejection [reviewed in [38]].

Infusion of host-type Th2.R cells one week pre-transplant optimally promoted host Th2-type immunity at the time of transplant and preserved allograft viability in 100% of recipients. Since the Th2.R cell product was generated in high-dose rapamycin, we predicted that the Th2.R cells may be relatively resistant to the effects of in vivo rapamycin, thereby further promoting an in vivo Th2 shift; however, to the contrary, co-administration of rapamycin drug therapy abrogated the ability of Th2.R cells to polarize host immunity towards a Th2 phenotype. In our previous murine studies, we did observe that ex vivo rapamycin resistance does not necessarily confer resistance to rapamycin in vivo [17]; such observations may be due to rapamycin blockade of non-T cell pathways in vivo, such as antigen-presenting-cell populations [39] that may help drive T cell expansion in vivo. Furthermore, we also determined that administration of rapamycin did not increase Foxp3^+^ regulatory T cells in vivo. In contrast to these results using in vivo rapamycin, we found that cyclosporine therapy was permissive for the immune modulatory effect generated through Th2 cell therapy. As such, although experimental models indicate that rapamycin has more tolerance-induction properties relative to cyclosporine therapy [40], [41], [42], our data indicate that calcineurin inhibitor therapy may be preferentially utilized in the setting of polarized Th2.R cell therapy for solid organ allografting. It is also important to note that infusion of Th2.R cells on the same day as cardiac allografting was sufficient to prolong allograft survival; this lack of requirement for pre-infusion of Th2 cells would be advantageous for clinical translation because of the unpredictability of cardiac allograft availability.

In conclusion, the adoptive transfer of ex vivo generated, rapamycin-resistant host Th2-type cells represents an effective new approach to the prolongation of cardiac allograft viability. These data support the current momentum in the field to develop adoptive cell therapies for tolerance induction in a calcineurin-inhibitor sparing manner, and suggests that functionally-defined and cytokine-polarized effector T cells, as well as Treg cells, may contribute to the future armamentarium to protect against solid organ allograft rejection.

## Materials and Methods

### Animal care

The experiments were performed as part of a protocol approved by the Animal Care and Use Committee (ACUC) of the Clinical Center of the National Institutes of Health (NIH; Animal study protocol approval number CCM 06-02). Inbred male brown Norway (BN) and dark agouti (DA) rats, weighing 200–250 g, were purchased from Charles River Laboratories (Wilmington, MA) and maintained under pathogen free conditions.

### Antibodies and reagents

RPMI media, rapamycin, and sodium pyruvate was obtained from Sigma (St.Louis, MO) and FCS was from Gem Cell (West Sacramento, CA). CD4 microbeads were from Miltenyi Biotec (Auburn, CA). Goat anti-rat Ig G was from Qiagen (Valencia, CA). Anti-CD3, anti-CD28 coated tosyl-activated magnetic beads were manufactured as previously described [43]. Recombinant human (rh) IL-2, (rh) IL-7 and (rr) IL-4 were from PeproTech (Rocky Hill, NJ). Penicillin-streptomycin-glutamine, nonessential amino acid and N-acetyl-L-cysteine was obtained from Invitrogen Life Technologies (Carlsbad, CA). All antibodies (unless otherwise stated) were purchased from BD Biosciences (BD; San Diego, CA); anti-rat Foxp3 APC was from eBioscience (San Diego, CA). Luminex kits for detection of rat IL-2, IL-4, IFN-γ and IL-10 were from Bio-Rad (Hercules, CA). Cyclosporine A (CSA or Sandimmune®) was from Novartis (Hanover, NJ).

### T cell subset isolation and ex vivo culture of Th2.R cells

Spleens were harvested from host type BN rats, RBC lysed and B cells were depleted (goat anti-rat IgG beads); CD4 T cells were enriched using Miltenyi CD4 microbeads as per manufacturer's instructions. Total CD4^+^ T cells were cultured in polystyrene tissue culture flasks (Corning; Lowell, MA). Cells were activated by anti-CD3 (clone:G4.18), anti-CD28 (clone: JJ316) co-stimulation (bead∶cell ratio, 3∶1) and cultured in RPMI media containing 10% FCS, 1× Sodium pyruvate, 1× non-essential amino acids, β-ME (5×10^−5^ M), 1× streptomycin-penicillin and glutamine. Cytokines (rh) IL-2 (100 IU/ml), (rh) IL-7 (20 ng/ml), (rr) IL-4 (10000 IU/ml) and rapamycin (10 µM) were added to the culture at day 0. Cultures were started at 1.5×10^6^ cells/ml, and (rh) IL-2 was again added at day 2. Cultures were harvested on day 3 for phenotyping and adoptive transfer experiments.

### Heterotropic cardiac transplantation

Brown-Norway (BN) rats served as recipient and Dark Agouti (DA) rats served as allograft cardiac donor. Allogeneic cardiac transplantations were performed using a modified version of the heterotopic cardiac transplantation model reported by Yokoyama *et al* [44]. As previously described [45], preparation of the donor heart for transplantation entailed ligation of pulmonary vessels, superior vena cava and inferior vena cava (IVC), creation of an atrial septal defect, and disruption of the tricuspid valve leaflets. The donor ascending aorta was anastamosed to the recipient abdominal aorta and the donor right atrium was anastamosed to the recipient IVC. Upon re-establishment of blood flow, all transplanted hearts resumed spontaneous contractions, had coordinated atrioventricular activity, and were free of gross surgical injury at the time of closure. The transplanted animals were subjected to various treatments with CSA (0, 10, 18, or 28 day regimen), rapamycin, and adoptive transfer of 1×10^7^ Th2.R cells per injection (at day 0 of transplant and/or day −7 pre-transplant), as summarized in Table 2. Survival of cardiac grafts was evaluated by daily palpation; cessation of heartbeat was interpreted as lack of viability. Specimens were processed for histopathology using hematoxylin and eosin staining [46].

**Table 2:** Summary of cardiac viability by palpation (day 28 post-transplant).

Treatment Cohort	Frequency of Isograft/Allograft Viability	Percent Allograft Viability	Median Graft Survival (Days)
1	10/10	100%	N/A
2	1/3	33%	24
3	0/7	0%	22
4	6/7	83%	N/A
5	0/7	0%	22.5
6	0/5	0%	20.5
7	0/4	0%	16.5
8	4/5	80%	N/A
9	7/7	100%	N/A
10	7/7	100%	N/A

1 = Isograft alone.

2 = Allograft alone.

3 = Allograft+CSA (10 days of therapy).

4 = Allograft+CSA (28 days of therapy).

5 = Allograft+Th2.R (Day 0 infusion).

6 = Allograft+Th2.R (Day −7 infusion).

7 = Allograft+Th2.R (Day −7+Day 0 infusion).

8 = Allograft+CSA (10 days)+Th2.R (Day 0 infusion).

9 = Allograft+CSA (10 days)+Th2.R (Day −7 infusion).

10 = Allograft+CSA (10 days)+Th2.R (Day −7+Day 0 infusion).

### Flow cytometry

T cells were washed with PBS supplemented with 0.1% BSA and 0.01% azide, and stained using anti-: CD4 PE-cy5 (clone ox-35), CD8 APC (clone ox-8), CD3 biotin (clone g.18), Streptavidin PE-cy7 and CD25 PE (clone ox-39). For intra-cellular (IC) flow cytometry, fixation and permeabilization buffer was utilized (eBioscience); Intracellular flow cytometry was performed with combinations of anti-: IFN-γ FITC (clone DB-1; Biolegend, San Diego, CA), IL-4 PE (clone ox-81), CD4 PE-Cy7 and Foxp3 Pacific Blue (clone FJK-16s; eBioscience). Cells were analyzed using a BD Biosciences LSR II (San Jose, CA) equipped with 488, 633, 405 and 355 nm lasers and thirteen detectors. Data was acquire using DiVa ver. 5.1 (BD Biosciences), and analyzed using FlowJo ver. 8.8 (Treestar Software, Ashland, WA).

### Allogeneic mixed lymphocyte reaction (MLR)

Bone marrow DC were generated from DA and BN rats, RBC lysed and cultured in RPMI media with 5% FCS, (rr) GM-CSF (50 ng/ml), and (rr) IL-4 (20 ng/ml) for 5 days. Bacterial LPS (1 µg/ml; Sigma) was added to the culture for 24 hrs and DC were harvested. The DC from syngeneic and allogeneic hosts were utilized to stimulate spleen cells for measuring allo-specific responses.

### Cytokine analysis using multibead array

The expanded Th2 cells were harvested at day 3 and extensively washed with media prior to restimulation with anti-CD3 and anti-CD28. Supernatants were collected 24 hr post-stimulation and analyzed for cytokines using the Biorad Multiplex bead array. Single cell suspensions of spleen (1×10^6^/ml), inguinal lymph nodes (1×10^6^/ml), and mesenteric lymph nodes (1×10^6^/ml) were obtained from rats post-transplant and stimulated with anti-CD3, anti-CD28 coated beads followed by incubation at 37°C for 24 hrs. The supernatants were harvested and Th1/Th2 cytokine profile was measured using a Biorad multiplex bead array. In some experiments, T cells were stimulated with BN or DA dendritic cells (DC) (spleen cell to DC ratio, 10∶1) for 24 h. Allogeneic cytokine secretion was subtracted out of syngeneic cytokine secretion and is presented as allo-specific responses.

### Statistical analysis

Flow cytometry and cytokine data were analyzed using student's 2-tailed *t* tests. Comparison values of p<0.05 were considered statistically significant. Survival was determined using Kaplan Meier test.
